# Thanksgiving

**Published:** 1890-10-11

**Authors:** 


### Words of Consolation.

### THANKSGIVING.

Our hospital wards have lately been receiving large hampers of fruit, vegetables, and flowers, sent from churches where harvest services have been held. Large clusters of grapes, rosy apples, great succulent green cabbages or golden marrows, and then masses of flowers and corn and grass. What a wondrous world it is that produces all this wealth and beauty! Is it not well to thank God for His goodness, and to heartily sing that His "mercy endureth for ever"? But some of those who are sick and sad can scarcely lift their thoughts to songs of thanksgiving, their own pains and griefs harrass their minds; they begin to doubt the goodness of God, for their lot is very bitter. Look, then, at the corn, examine the flowers—every leaf and petal has a thousand veins, and how marvellously the husks enclose and shelter the swelling grain. Oh ye of little faith! God so clothed the grass of the field; shall He then forget His suffering, sentient children?

It is a fit thing that the harvest decorations should be forwarded for the use of those who are ill in hospitals; the fruit is so welcome to feverish lips, the flowers so beautiful to weary eyes. Human sympathy is very sweet, but the lips of men speak coarsely and jarringly compared with the soft messages the flowers can bring. "God so clothed the grass of the field"; look at it closely this grass of the field which is cut down every year; notice the wondrous workmanship of the very smallest part. "God so clothed the grass of the field." And can you doubt then, in spite of all the pain and grief, that He careth for you, is very near you, and will shortly grant you your heart's desire? It may be impossible to see the reason for present sorrows, but they become very bearable if we can believe that God cares for us, that it is His hand which sends the suffering. Try and lie still; try and draw nearer to Christ, and perhaps you will be able to see that it is all for the best. If God clothed so carefully every blade of the grass growing over this great world, shall He not be equally careful in tending you, His child?

